# Factors of presenteeism and its association with detrimental effects among employees in Switzerland working in different sectors – a cross-sectional study using a multi-item instrument

**DOI:** 10.1007/s00420-024-02083-x

**Published:** 2024-07-01

**Authors:** Maisa Gerlach, Eva Blozik, André Meichtry, Miriam Hägerbäumer, Gablu Kilcher, Christoph Golz

**Affiliations:** 1 Department of Health Professions, Bern University of Applied Sciences, Murtenstrasse 10, Bern, 3008 Switzerland; 2 Department Health Services Research, SWICA Health Organization, Winterthur, Switzerland; 3 Department of Psychology, EURO-FH University of Applied Sciences, Hamburg, Germany

**Keywords:** Presenteeism, Culture, Chronic disease, Regression, Swiss employees, Health outcomes

## Abstract

**Purpose:**

Presenteeism, the phenomenon of employees working despite illness, is a significant issue globally, impacting individual well-being and organizational efficiency. This study examines presenteeism among Swiss employees, exploring its occurrence, primary factors, reasons, and impact on employees’ health.

**Methods:**

This study used cross-sectional data from 1,521 employees in different sectors in Switzerland. Descriptive statistics and multiple linear models for influencing factors and detrimental effects, such as burnout symptoms, job satisfaction, general health, and quality of life, were calculated for data analysis. Presenteeism was measured using the Hägerbäumer multi-item scale, ranging from 1 = “Never in case of illness” – 5 = “Very often in case of illness.”

**Results:**

The employees reported that in case of illness, they rarely worked in the last 12 months M = 2.04 (SD = 1.00). A positive approach to presenteeism in the team was associated with less presenteeism (β = -0.07) and problematic leadership culture in dealing with presenteeism with increased presenteeism (β = 0.10). In addition to well-known factors, presenteeism was significant for burnout symptoms (β = 1.49), general health status (β = -1.5), and quality of life (β = -0.01).

**Conclusion:**

The study offers insights into the phenomenon of presenteeism among Swiss employees in various sectors by applying a multi-item scale for presenteeism. The findings indicate that a positive team dynamic and organizational culture may significantly reduce presenteeism. Presenteeism behavior is a significant factor of adverse outcomes. This highlights the importance of acknowledging presenteeism in the context of occupational health.

**Supplementary Information:**

The online version contains supplementary material available at 10.1007/s00420-024-02083-x.

## Introduction

Presenteeism, the phenomenon of employees working despite illness, is widespread and affects workers worldwide, with prevalence rates ranging from 30% to over 90% (Chambers et al. 2017; Lohaus and Habermann 2020; Min et al. 2022). This issue has prompted growing attention towards employee health, due to its significant impact on personal well-being and organizational efficiency (Lohaus and Habermann 2019; Miraglia and Johns 2016; Ospina et al. 2015; Ruhle et al. 2019). Studies indicate that presenteeism can result in greater productivity losses than absenteeism (Evans-Lacko and Knapp 2016; Kigozi et al. 2017), exemplified by cases like Switzerland, where, in 2016, it accounted for about two-thirds of health-related production losses, nearly tripling the costs of absenteeism (Igic et al. 2017). Presenteeism refers to the behavior of employees who work despite being ill, which would normally warrant an absence (Ruhle et al. 2019). The decision to work despite illness is complex, influenced by individual, job, and organizational factors (Lohaus and Habermann 2019, 2020), and is especially prevalent in professions like healthcare and education (Al Nuhait et al. 2017; Gustafsson et al., 2013; Martinez and Ferreira 2012). Modern working conditions, including remote work, have further nuanced presenteeism behaviors (Breitsohl et al. 2023; Lohaus and Habermann 2020; Priebe and Hägerbäumer 2023). Common reasons include feelings of irreplaceability, workload pressures, and a reluctance to inconvenience colleagues (Marklund et al. 2021).

Presenteeism can have significant implications for both the employee and the company. These consequences have been thoroughly studied and documented (Aronsson and Gustafsson 2005; Banks and Pearson 2021; Johns 2010). For the company, presenteeism can lead to productivity losses and substantial costs. Meanwhile, employees may experience long-term adverse health consequences, such as worse health status, lower mental well-being, emotional exhaustion, or higher rates of depression (Lohaus and Habermann 2019).

Traditional single-item measures of presenteeism have limitations in validity and reliability (Diamantopoulos et al. 2012), prompting a shift towards multi-item scales like the Hägerbäumer scale (Ruhle et al. 2019), which, however, lacks comprehensive application in research outside its development and validation contexts (Hägerbäumer 2017).

This study aims to examine the prevalence and underlying reasons for presenteeism among Swiss employees, (1) the occurrence of presenteeism across various sectors, (2) the reasons behind presenteeism, (3) the primary factors linked to presenteeism among employees working in different sectors, and (4) the association between presenteeism and health outcomes with the multi–item Hägerbäumer presenteeism scale.

## Method

### Design

This study is based on a cross-sectional study design and is part of the project “Occupational Health Management and Presenteeism among Swiss Employees “(Presenteeism at Work 2021). The quasi-experimental project consists of two data measurements (T^0^, T^1^) from 2021 to 2023. Between the measurements, an e-learning intervention was conducted. In this study, the results of the baseline measurement (T^0^) are reported. We adhered to the STROBE reporting guideline for cross-sectional studies (Von Elm et al. 2007). The checklist can be found as supplementary file A.

### Recruitment

A convenience sampling among companies from the German-speaking part of Switzerland was conducted. The companies were identified from national associations’ lists of working sectors, such as the construction industry, healthcare or education. The Chief Executive Officers or the head of Human Resources received information about the project by email or telephone. The email comprised a flyer and a short film containing information about the project. The participating companies differed regarding their size, categorized as small (10–49 employees), medium (50–249 employees), and large (over 250 employees). A total of 16 companies in the German-speaking part of Switzerland took part in this study (small = 5; medium = 6; large = 5).

### Study sample and data collection

For data collection, one contact person in each participating company was responsible for distributing the questionnaires. The questionnaire was sent to all employees in the company. They were informed about the study using a short film and a written study flyer. The questionnaire was available in German and English, both online via Unipark®. Participants had one month to complete the questionnaire and received a reminder after two weeks. Each participant generated a unique code based on the first three letters of the mother’s and father’s names and their birth months. This allowed the identification of unique cases.

### Questionnaire

**Fig. 1:**
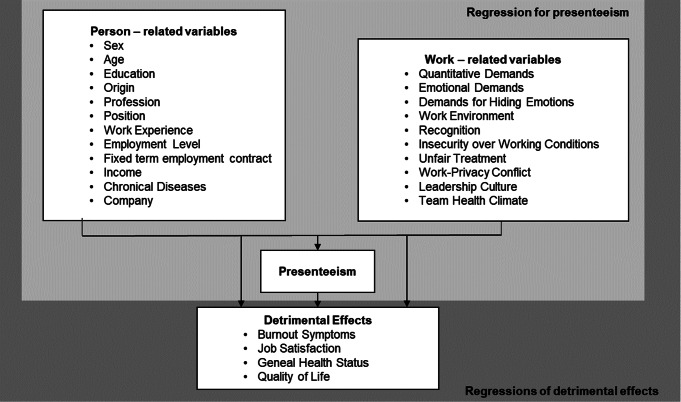
Analysis model for multiple linear regression models

For this study, a questionnaire was developed based on the model “Research framework for the content of a decision-integrated model of presenteeism” by Lohhaus and Habermann (2019) (Fig. 1). The following valid and reliable scales with a Cronbach’s alpha (α) between 0.6 and 0.8 from the Copenhagen Psychosocial Questionnaire (COPSOQ) (Burr et al. 2019) were used to measure work-related factors of presenteeism: Quantitative demands, emotional demands, hiding emotions, appreciation, insecurity working conditions, work-family conflict, and work environment. All item responses of the COPSOQ were scored on a five-point Likert scale ranging from always - never/hardly never or to a very large extent - to a very small extent, with a high score indicating high demands. The Hägerbäumer Presenteeism Scale (α = 0.89) for measuring presenteeism as a behavior ranges between 1 = “Never in case of illness” – 5 = “Very often in case of illness” (Hägerbäumer 2017), with a high value corresponding to frequent presenteeism. The Presenteeism Climate Questionnaire (α = 0.89) ranges between 0= “completely disagree” − 7 = “totally agree” with a high value indicating problematic leadership culture in dealing with presenteeism (Ferreira et al. 2015). The Team Health Climate Questionnaire (α = 0.71) ranges between 1= “disagree” − 4= “agree” with a higher value for positive handling in the team regarding presenteeism (Schulz et al. 2017).

As detrimental effects, the General health status – EQ VAS (0= “The worst health you can imagine” – 100 = “The best health you can imagine”), the quality of life questionnaire EQ-5D – 5 L (EuroQol Research Foundation, 2019) and the COPSOQ scales job satisfaction and burnout-symptoms (Burr et al. 2019) were measured. The EQ-5D-5 L (α = 0.85) assesses an individual’s health and quality of life by evaluating five dimensions: mobility, self-care, usual activities, pain, and depression. Respondents choose from five response levels for each dimension, ranging from 1 = “no problems” to 5 = “extreme problems” to describe their health status (EuroQol Research Foundation, 2019).

To identify the most important reasons employees chose presenteeism, we adopted the reasons examined by Hägerbäumer (2017). We developed four items of our own (according to the latest results of the Swiss State Secretariat for Economic Affairs (SECO): (1) I did not work on-site, but remotely/Home Office; (2) Because I enjoy my job; (3) I did not want to stay at home. All items could be answered on a five-point Likert scale ranging from 1 (Almost Never True) to 5 (Almost Always True). The questionnaire also included socio-demographic questions such as sex, age, education or company (Fig. 1).

### Analysis

Data was analyzed using R 3.6.0 (R Core Team 2021). In case of duplicates, only the first completed questionnaire was included. In case of missings, we computed listwise deletion. For data analysis, the COPSOQ scales were transformed to a value range from 0 (minimum value) to 100 points (maximum value). No average score was calculated if less than half of the questions in a scale had been answered (Kristensen, 2005). Mean scores were calculated for the COPSOQ scales, the Hägerbäumer Presenteeism scale, the Presenteeism Climate Questionnaire, and the Team Health Climate Questionnaire. Participants who reported that they had not been ill for the last 12 months were excluded from the mean score calculation of the Hägerbäumer Presenteeism scale (Hägerbäumer 2017). The EQ-5D-L5 quality of life questionnaire was calculated using the standard EQ-5D-5 L index values as defined in the EuroQol Group guidelines for Germany (EuroQol Research Foundation, 2019). The variable company was categorized into sectors according to the sector structure given by the Swiss Federal Statistical Office.

First, descriptive statistics regarding the study sample, as well as the extent of presenteeism among different sectors in Switzerland, were computed. Second, reasons for presenteeism among employees were analyzed using descriptive statistics. Third, we fitted a multiple linear model to the outcome variable ‘Hägerbäumer Presenteeism’ with all independent variables shown in Fig. 1. Fourth, we computed multiple linear regressions for the detrimental effects: Burnout symptoms, Job Satisfaction, General Health Status, and Quality of Life to elaborate the association with presenteeism, besides known relevant associated factors shown in Fig. 1. The final model was computed with a stepwise backward algorithm (R package MASS, function stepAIC) using the Akaike information criterion (AIC). Residual analysis was performed to assess model assumptions, and the Variance Inflation Factor was computed for testing multicollinearity. In the case of evidence of heteroscedasticity, we computed robust standard errors (Zeileis et al. 2019).

## Results

The study sample consisted of 2,183 employees from 16 companies. Overall, 662 participants mentioned that they were not ill in the last 12 months (30.3%) and were excluded from further analysis. This resulted in a sample of 1,521 employees who reported being ill at least once in the last 12 months (69.7%). Most participants worked in the insurance sector (65.6%). Most participants were female (64.2%) with a mean age of 50.0 years (SD = 11.9); they had an average of 12.3 (SD = 10.2) years of professional experience and 7.8 (SD = 7.1) years working in their current company. Most participants originated from Switzerland (84%) (see Table 1).

**Table 1:** Sample characteristics

Characteristics	Mean (SD)	*N* (%)
Age	50.0 (11.9)	
*Sex*		
Female		1013 (66.6)
Male		508 (33.4)
Education		
No education		14 (0.9)
Secondary II		555 (36.5)
Tertiary B		267 (17.6)
BSc		150 (9.9)
MSc		111 (7.3)
PhD		36 (2.4)
Missing		388 (25.5)
Professional experience	12.3 (10.2)	
Current position (years)	7.8 (7.1)	
Income (annual) in CHF	78’857.5 (94’553.8)	
*Sector*		
Insurance		1002 (65.9)
Healthcare		153 (10.0)
Education		110 (7.1)
Informatics		95 (6.2)
Social Services		94 (6.1)
Manufacture		54 (3.6)
Production of printed products		10 (1)
Gastronomy		3 (0.1)
*Origin*		
Switzerland		1280 (84.2)
Other countries		241 (15.8)

### The extent of presenteeism among different sectors.

**Fig. 2:**
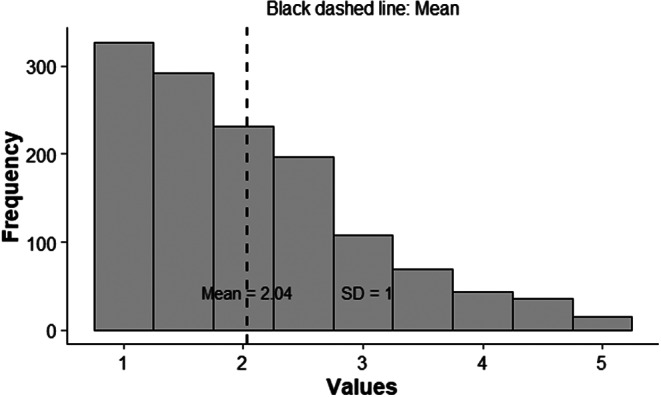
Histogram showing the Distribution of Presenteeism

The participants reported that in case of illness, they rarely worked in the last 12 months, with a mean of 2.04 (SD = 1.00). In Fig. 2 the distribution of presenteeism is shown in a histogram.

Among the six items of the Hägerbäumer scale, the item “I came to work despite illness” showed the highest mean with 2.4 (SD = 1.2). The sector “Production of printed products” sector had the highest values among the possible items, although with a small number of participants (see Table 2).

### Results on reasons for presenteeism

In Table 3, the reasons for presenteeism per sector are summarized. The most relevant reasons for presenteeism across sectors were “I had too much to do” and “there was urgent work to do and appointments”, with a mean of 3.2 (SD = 1.4). Across the sectors, the participants working in education reported on five of the 13 possible reasons for the highest values.

**Table 2:** Descriptives on scale and item level for hägerbäumer presenteeism scale

	Total	Sectors							
		Insurance	Healthcare	Education	Informatics	Social Services	Manufacture	Production of printed products	Gastronomy
	Mean (SD)*	Mean (SD)*	Mean (SD)*	Mean (SD)*	Mean (SD)*	Mean (SD)*	Mean (SD)*	Mean (SD)*	Mean (SD)*
Hägerbäumer Presenteeism Scale	2.04 (1.00)	2.08 (1.00)	2.07 (0.87)	**2.16 (0.87)**	1.80 (0.85)	1.81 (0.82)	2.01 (0.97)	2.12 (1.43)	1.83 (1.04)
*Items*									
I came to work despite illness.	2.42 (1.20)	2.42 (1.22)	2.48 (1.11)	**2.71 (1.11)**	2.20 (1.15)	2.14 (1.06)	2.55 (1.30)	2.60 (1.58)	1.67 (0.58)
I worked even though my doctor advised against it.	1.54 (0.99)	1.57 (1.04)	1.51 (0.88)	1.58 (0.89)	1.38 (0.81)	1.40 (0.81)	1.59 (1.06)	**2.20 (1.75)**	1.67 (0.58)
I worked in spite of showing more severe symptoms of illness (e.g., pain, chills, fever).	1.82 (1.11)	1.89 (1.15)	1.69 (0.98)	1.78 (1.04)	1.68 (1.02)	1.49 (0.95)	1.90 (1.05)	**2.40 (1.71)**	1.33 (0.58)
I worked the full working day or the full shift despite illness.	2.12 (1.19)	2.15 (1.22)	2.21 (1.13)	2.17 (1.12)	1.95 (1.09)	1.87 (1.04)	2.04 (1.20	**2.40 (1.65)**	1.33 (0.58)
Due to acute health problems, I took medication in order to be able to work.	2.36 (1.28)	2.40 (1.30)	2.44 (1.21)	2.56 (1.23)	1.89 (1.11)	2.07 (1.11)	2.25 (1.30)	2.44 (1.51)	**2.67 (2.08)**
Although I was ill, I dragged myself to work.	2.17 (1.19)	2.18 (1.23)	2.22 (1.04)	2.40 (1.12)	1.89 (1.11)	1.87 (1.08)	2.21 (1.21)	**2.40 (1.71)**	2.33 (2.31)

*Hägerbäumer Presenteeism scale ranges between 1 (never in case of illness) and 5 (very often in case of illness)

**Table 3:** Reasons for presenteeism

	Total	Sectors							
		Insurance	Healthcare	Education	Informatics	Social Services	Manufacture	Production of printed products	Gastronomy
	Mean (SD)*	Mean (SD)*	Mean (SD)*	Mean (SD)*	Mean (SD)*	Mean (SD)*	Mean (SD)*	Mean (SD)*	Mean (SD)*
I didn’t want to be a burden to my colleagues.	3.0 (1.5)	3.0 (1.5)	3.7 (1.4)	3.0 (1.4)	2.6 (1.4)	3.0 (1.5)	2.8 (1.5)	3.7 (1.8)	**5.0 (0.0)**
There was no replacement for me.	2.6 (1.5)	2.4 (1.5)	3.2 (1.4)	3.6 (1.3)	2.5 (1.5)	2.8 (1.4)	2.5 (1.5)	**3.8 (1.6)**	1.5 (0.7)
There was urgent work to do and appointments.	3.2 (1.4)	3.1 (1.5)	3.1 (1.4)	**4.1 (0.9)**	3.1 (1.5)	3.2 (1.3)	3.1 (1.4)	3.9 (1.6)	1.0 (0.0)
I had too much to do.	3.2 (1.4)	3.2 (1.4)	3.0 (1.4)	**3.8 (1.1)**	2.9 (1.5)	3.1 (1.3)	3.0 (1.3)	3.5 (1.4)	2.7 (1.5)
I felt capable enough to do it.	3.0 (1.2)	2.9 (1.2)	2.9 (1.1)	3.1 (0.9)	2.8 (1.4)	3.1 (1.1)	2.8 (1.1)	2.9 (1.1)	**4.0 (0.0)**
The work would have piled up.	3.0 (1.4)	3.0 (1.5)	2.5 (1.4)	**3.6 (1.2)**	2.8 (1.4)	2.9 (1.3)	3.0 (1.4)	3.3 (1.7)	2.0 (1.4)
All my colleagues work when they are sick.	2.1 (1.0)	2.0 (1.0)	**2.5 (1.1)**	**2.5 (1.0)**	1.8 (1.0)	2.1 (0.9)	1.8 (1.0)	2.2 (0.9)	1.5 (0.7)
I wanted to show the company my positive attitude.	2.2 (1.3)	2.3 (1.3)	2.1 (1.3)	2.1 (1.2)	2.1 (1.3)	2.1 (1.2)	2.3 (1.3)	2.4 (1.4)	**3.0 (2.8)**
I was afraid of professional disadvantages if I missed work.	1.8 (1.2)	**1.9 (1.2)**	1.6 (1.0)	1.7 (1.0)	1.7 (1.1)	1.7 (1.1)	1.7 (1.1)	1.6 (1.0)	1.0 (0.0)
I needed distraction from my illness.	1.5 (0.9)	1.6 (1.0)	1.4 (0.7)	1.3 (0.7)	1.6 (1.1)	1.4 (0.8)	**1.7 (1.1)**	1.6 (0.8)	1.0 (0.0)
I did not work on-site, but remotely/Home Office.	3.0 (1.6)	3.3 (1.5)	1.2 (0.5)	**3.5 (1.4)**	3.2 (1.5)	2.0 (1.3)	2.6 (1.6)	2.5 (1.4)	1.0 (0.0)
Because I enjoy my job.	2.7 (1.3)	2.7 (1.3)	2.7 (1.3)	2.9 (1.1)	2.7 (1.4)	2.9 (1.3)	**2.8 (1.3)**	2.5 (1.4)	2.0 (1.4)
I didn’t want to stay at home.	1.6 (1.1)	1.6 (1.1)	**1.9 (1.2)**	1.3 (0.6)	1.5 (0.9)	1.8 (1.1)	1.5 (1.1)	1.7 (1.3)	1.0 (0.0)

*Reasons for presenteeism range between 1 (Almost Never True) and 5 (Almost Always True)

### Results of the multiple linear regression model for presenteeism

Table 4 summarizes the results of the multiple linear regression model for presenteeism. The model explained 32% of the variance: R2 = 0.32, the overall F-test resulted in F(23,861) = 17.41, *p* < .001. Being male was found to be associated with reduced presenteeism (*B* = -0.39, *p* < .001). Age was positively associated with presenteeism (*B* = 0.02, *p* < .001), representing a 0.02-point increase in presenteeism per additional year. A positive approach to presenteeism in the team was associated with less presenteeism (*B* = -0.07, *p* = .08) and a problematic leadership culture in dealing with presenteeism with increased presenteeism (*B* = 0.10, *p* < .001). Further information about the associations of the factors with presenteeism is given as Added-Variable Plots (Partial Regression Plots) in the supplementary files A-C.

**Table 4:** Multiple linear regression with presenteeism as an outcome

Coefficient	β	B	Std. Error	T-value	*p*-value		CI (95%)
Intercept	0.41	1.05	0.25	4.19	< 0.001		0.56–1.54
Sex: Male	-0.42	-0.39	0.06	-6.11	< 0.001		-0.51 - -0.27
Age	0.22	0.02	0.003	6.88	< 0.001		0.01–0.02
Education: No Education	-0.36	-0.34	0.24	-1.42	0.16		-0.71–0.04
Education: Master of Science	-0.20	-0.19	0.10	-1.81	0.07		-0.37 - -0.01
Temporary employment contract: Yes	-0.14	-0.13	0.08	-1.59	0.11		-0.29–0.03
Employment Level	-0.05	-0.01	0.008	-1.52	0.13		-0.03–0.002
Annual gross income in CHF	-0.20	-0.18	0.06	-2.90	0.004		-0.32 - -0.05
Quantitative Demands	0.09	0.005	0.002	2.64	0.008		0.001–0.008
Work-Privacy Conflict	0.25	0.01	0.002	6.64	< 0.001		0.007–0.014
Unfair Treatment	0.09	0.004	0.002	2.56	0.01		0.001–0.008
Leadership Culture	0.13	0.10	0.03	3.87	< 0.001		0.05–0.15
Team Health Climate	-0.06	-0.07	0.04	-1.73	0.08		-0.15–0.01
Chronic Disease: Musculoskeletal condition	0.19	0.18	0.10	1.72	0.09		-0.04–0.40
Chronic Disease: Mental	-0.31	-0.29	0.15	-1.91	0.06		-0.52 - -0.05
Chronic Disease: Digestive System	0.57	0.53	0.15	3.45	< 0.001		0.21–0.86
Chronic Disease: Tumors/Cancer	1.06	0.99	0.37	2.70	0.007		0.05–1.92
Sector: Gastronomy	-0.43	-0.40	0.79	-0.51	0.61	0.03	-0.67- -0.13
Sector: Healthcare	-0.27	-0.26	0.15	-1.75	0.08		-0.52–0.01
Sector: Production of printed products	-0.05	-0.05	0.29	-0.16	0.87		-0.67–0.57
Sector: Manufacture	0.14	0.13	0.19	0.67	0.50		-0.25–0.50
Sector: Informatics	-0.26	-0.24	0.17	-1.46	0.14		-0.53–0.05
Sector: Social Services	0.03	0.03	0.16	0.19	0.85		-0.26–0.32
Sector: Insurance	0.01	0.01	0.13	0.09	0.93		-0.24–0.27

Hägerbäumer Presenteeism scale ranges between 1 (never in case of illness) and 5 (very often in case of illness)

Reference category for sectors: Education

Standardized (β) and unstandardized (B) regression coefficients

### Results of the multiple linear regression models for detrimental effects

Table 5 summarizes the results of the multiple linear regressions for the detrimental effects. Presenteeism was a significant factor of all detrimental effects, except for job satisfaction, when considering other relevant work-related factors. Higher presenteeism led to more burnout symptoms (*B* = 1.49) and lower general health status (*B* = -1.50), as well as lower quality of life (*B* = -0.01).

**Table 5:** Multiple linear regression models with detrimental effects as outcomes

		Burnout symptoms			Job satisfaction			General Health Status			Quality of Life		
		*R*^*2*^ *= 0.49, F(17,867) = 51.35, p < .001*			*R*^*2*^ *= 0.49, F(19,863) = 43.26, p < .001*			*R*^*2*^ *= 0.25, F(13,871), 22.28, p < .001*			*R*^*2*^ *= 0.34, F(23,852), 19.04, p < .001*		
		β	B	se	β	B	se	β	B	se	β	B	se
Intercept		-0.08	8.56°	4.46	0.31	68.84***	2.62	0.05	84.11***	2.18	0.45	1.00***	0.02
Sex: Male		-0.19	-3.82**	1.17	0.08	1.25	0.88	0.10	1.42	0.91			
Age		0.06	0.11°	0.06									
Education: No Education					0.56	8.33**	3.16						
Education: Bachelor of Science											-0.13	-0.01	0.01
Education: Master of Science		-0.20	-3.95*	1.88									
Middle management level		-0.14	-2.75	1.76	0.13	1.98	1.31						
Work Experience		0.05	0.11°	0.06									
Annual gross income in CHF		0.09	1.89	1.17									
Temporary employment contract: Yes		0.11	2.31	1.48									
Employment Level		-0.07	-0.35*	0.14									
Quantitative Demands		0.08	0.085**	0.03				-0.05	-0.04	0.03			
Emotional Demands											0.08	0.0002*	0.0001
Demands for Hiding Emotions		0.11	0.086***	0.02									
Work Environment		0.11	0.13***	0.03							-0.08	-0.0004*	0.0001
Recognition					0.29	0.19***	0.02	0.11	0.07***	0.02	0.06	0.0002°	0.0001
Insecurity over Working Conditions					-0.07	-0.05*	0.22	0.09	0.06*	0.03	0.09	0.0004**	0.0002
Work-Privacy Conflict		0.47	0.45***	0.03	-0.19	-0.13***	0.02	-0.22	-0.14***	0.03	-0.23	-0.001***	0.0001
Unfair Treatment					-0.21	-0.16***	0.02	-0.06	-0.05°	0.03	-0.12	-0.0006***	0.0002
Team Health Climate					0.13	2.71***	0.56						
Leadership Culture		0.08	1.38**	0.45	-0.09	-1.16**	0.36	-0.12	-1.39***	0.40	-0.17	-0.013***	0.003
Presenteeism		0.07	1.49*	0.61	-0.04	-0.64	0.45	-0.10	-1.50**	0.51	-0.13	-0.01***	0.003
Chronic Disease: Accident Injuries											-0.95	-0.09***	0.04
Chronic Disease: musculoskeletal system								-0.35	-4.94**	1.58	-0.60	-0.06***	0.01
Chronic Respiratory disease											0.26	0.02	0.02
Chronic Disease: Mental		0.39	7.84**	2.72	-0.34	-5.01*	2.06	-0.70	-9.94***	2.36	-0.60	-0.06***	0.01
Chronic Disease: Tumour/Cancer								-0.36	-11.73*	5.64			
Chronic Disease: Urogenital Tract		0.57	11.54°	5.94									
Chronic Disease: Congenital		0.59	11.94	7.31									
Chronic Disease: Blood					0.42	6.31°	3.37				-0.39	-0.05*	0.02
Chronic Disease: Neurological and sensory								-0.83	-5.11*	2.17	-0.24	-0.04**	0.01
Chronic Disease: Skin											-0.57	-0.02	0.01
Chronic Disease: Other								-0.33	-4.70*	1.98			
Sector**	Gastronomy				-2.57	-38.39***	10.96				-0.29	-0.03	0.08
	Healthcare				-0.33	-4.92*	1.94				-0.25	-0.02	0.01
	Production of printed products				-0.40	-5.96	4.01				-0.95	-0.09**	0.03
	Manufacture				-0.43	-6.47*	2.54				-0.19	-0.02	0.02
	Informatics				-0.43	-6.47**	2.22				-0.16	0.01	0.02
	Social Services				-0.32	-4.77*	2.12				-0.28	-0.03°	0.02
	Insurance				-0.38	-5.62**	1.74				-0.40	-0.04**	0.01

Significance level: °*p* < .1; *p ≤ .05; ** *p* < .01; *** *p* < .001; standardized (β) and unstandardized (B) regression coefficients; *se*: standard errors

^a^Mean score range from 0 (do not agree at all) to 100 (fully agree)

^b^Score range from 0 (The worst health you can imagine) to 100 (The best health you can imagine)

Reference category for sectors: Education

## Discussion

This study presents findings on presenteeism behavior measured with the Hägerbäumer scale among Swiss employees in various sectors, including the relationships between different factors, detrimental effects, and reasons for presenteeism. Overall, the participants reported moderate presenteeism, which goes in line with the findings of a German study that came to comparable results but only included the healthcare sector (Hägerbäumer 2017).

The results show that although the global test was significant, there are no significant differences between the sectors included and that employees exhibit presenteeism, albeit infrequently. This outcome contradicts other studies, which identified differences between sectors of blue vs. white-collar employees (Böckerman and Laukkanen 2010; Gustafsson and Marklund 2011; Marklund et al. 2021). One reason for this discrepancy could be the underrepresentation of blue-collar workers in our sample, with manufacturing, production of printed products, and gastronomy accounting for only 4.7% of the sample. The difference may further be attributed to the different measurements of presenteeism in our study compared to others. We used a multi-item questionnaire scale to measure presenteeism. The questionnaire captures various aspects of presenteeism behavior, enabling the construct to be recorded differently. It does not solely measure the frequency of absence from work in the last 12 months. This approach can produce more precise and accurate participant responses (Ruhle et al. 2019). To elaborate on this discrepancy, further research should compare single-item and multi-item presenteeism behavior scales regarding their convergent and discriminant validity.

The reasons for presenteeism in this study are similar to those found in other studies (Al Nuhait et al. 2017; Gustafsson Sendén et al. 2013; Hansen and Andersen 2008; Marklund et al. 2021). In our study, people reported that they often went to work because they had a huge workload, urgent appointments and work to do. A possible reason for this could be that a larger proportion of our sample works in white-collar sectors such as assurance. In this study, three reasons were added as possibility to report for presenteeism behavior. Although not the highest, working remotely or in home office was found to be prevalent among employees working in white collar sectors. COVID-19 in particular has led to a major change in working conditions and promoted working from home and remote. The changed conditions are increasingly being considered in presenteeism research, as research indicates that a low ability to disengage from work and low support from the supervisor are associated with a higher number of presenteeism behavior from remote. Remote working conditions thus appear to encourage presenteeism (Schmitz et al. 2023). This finding underlines the need for further development in the measurement of presenteeism, as the wording of the Hägerbäumer presenteeism scale implies work on site (Priebe and Hägerbäumer 2023).

In terms of factors associated with presenteeism, our findings suggest that a positive approach to taking the time needed to recover within the team can help to reduce presenteeism. This is underlined by the fact that a significant factor contributing to presenteeism is when there is a pervasive culture within an organization that values and rewards long hours and constant presence at work (Webster et al. 2019). This culture can pressure employees to come to work even when they are unwell. They may fear that taking time off sick will be perceived as a lack of commitment or dedication to their job. In such an environment, employees may feel compelled to come to work regardless of their health.

Furthermore, the results indicate that presenteeism may be influenced by sex and age. Specifically, the study found that male and younger employees experience less presenteeism compared to their older and female counterparts in our research. There are conflicting results regarding this finding (Webster et al. 2019). Some studies show the opposite, with men being more susceptible and other females (Robertson et al. 2012; Taloyan et al. 2016). Further, other studies report that younger employees are more affected, while others suggest that older employees are (d’Errico et al. 2016; Susser and Ziebarth 2016). Thus, the findings in this study on the association of demographic variables with presenteeism should not be overestimated, which is underlined by a meta-analysis that reported weak associations between demographic variables and presenteeism (Miraglia and Johns 2016).

Considering other well-known and relevant work-related factors for detrimental effects, we confirmed that presenteeism is a relevant factor of burnout symptoms, general health, and quality of life. Hägerbäumer (2020) argues that this association may be relevant for preventive measures, as presenteeism can be seen as an indicator for self-harming health behaviour. Therefore, presenteeism should be considered a significant factor in occupational health. The fact that something is changing in this respect is shown, for example, by including presenteeism as a single item in the latest version of the COPSOQ (Lincke et al. 2021), a popular instrument for research and risk assessment of workplace psychosocial conditions worldwide. However, the application of different instruments again limits the international comparability of results.

### Strengths and limitations

This article is based on the STROBE reporting guideline for cross-sectional studies (Von Elm et al. 2007). The study further uses a large sample size. This enabled us to conduct robust statistical analyses and gain deeper insights into our research questions. The large sample size allowed us to identify associations in the data more precisely and minimize the likelihood of random results, contributing to the internal validity of our study. Another strength of this study is its use of valid and reliable measurement instruments and scales, in particular the use of a multi-item scale for measuring presenteeism, which has not been applied to this extent to date.

Despite these strengths, our study has several limitations that must be considered. One major limitation is that our sample is not representative of the population as a whole, which means that the results may not be easily transferable to the broader population. On one hand, the study included companies that are receptive to the topic, potentially resulting in lower presenteeism or a more positive corporate culture. On the other hand, the voluntary nature of the questionnaire may have led to underrepresentation of certain employee groups. Future research should, therefore, aim to include a more diverse sample to increase the generalizability of the results. It is important to note that our results may not be directly comparable to those of other studies due to the use of specific measurement methods and scales. This may make it difficult to compare our research with studies that use different instruments. Therefore, when interpreting our results and future research, it is essential to consider this limitation to draw consistent and meaningful conclusions. Overall, these strengths and constraints emphasize the significance of interpreting our results carefully and the necessity for further research to address the limitations above and enhance understanding in this area of research.

## Conclusions

In conclusion, this study offers valuable insights into the phenomenon of presenteeism among Swiss employees in various sectors. The findings illuminate the relationships between different factors, the detrimental effects of presenteeism, and the reasons why employees decide to come to work while unwell. Our findings suggest that a positive team dynamic and organizational culture may significantly reduce presenteeism. A workplace culture that places a high value on long working hours and constant presence may contribute to presenteeism by pressuring employees to come to work even when unwell. Employees may attend work despite their health issues because they fear being perceived as less committed or dedicated. An organization’s values and culture are critical because they influence presenteeism.

We utilized the multi-item Hägerbäumer scale to assess presenteeism, focusing on the various aspects of presenteeism behavior allows for a more nuanced recording of the construct, rather than solely relying on the frequency of absences from work in the past 12 months, which is an advancement in the field and offers a pioneer database for comparison in future research.

Presenteeism behavior is a significant factor of detrimental effects, such as burnout symptoms, general health issues, and overall quality of life. This highlights the importance of acknowledging presenteeism in the context of occupational health. Considering these findings, it is essential to regard presenteeism as a significant factor in employees’ overall well-being and investigate its impact on different sectors and demographics.

## Electronic supplementary material

Below is the link to the electronic supplementary material.

Supplementary Material 1

Supplementary Material 2

Supplementary Material 3

## Data Availability

The raw dataset analyzed in the current study is available from the corresponding author upon reasonable request.
