# Supplementary figures and images for: Factors of presenteeism and its association with detrimental effects among employees in Switzerland working in different sectors – a cross-sectional study using a multi-item instrument
Source: Int Arch Occup Environ Health. 2024 Jul 1;97(7):767–78. doi: 10.1007/s00420-024-02083-x (PMC11416405; doi:10.1007/s00420-024-02083-x)

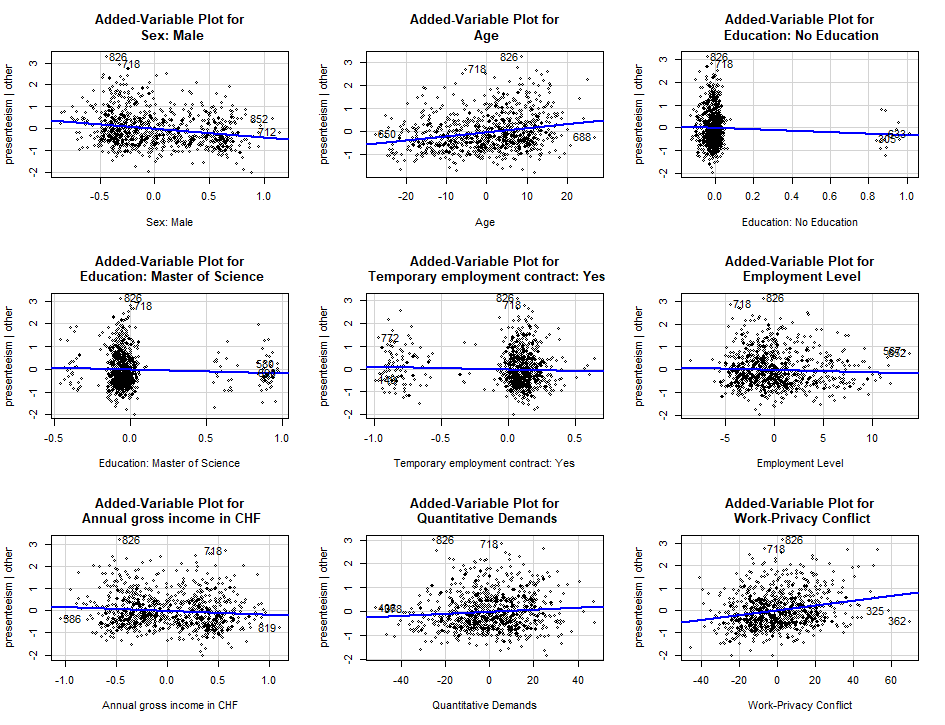

Supplement: Supplementary file 1 — Supplementary Material 1 [file 420_2024_2083_MOESM1_ESM.png]

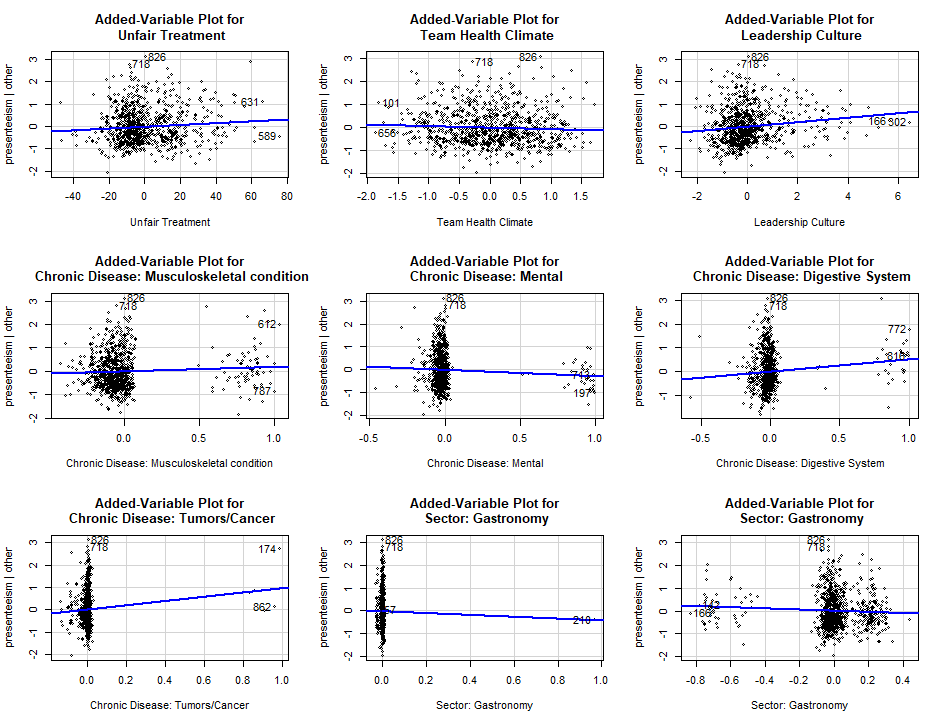

Supplement: Supplementary file 2 — Supplementary Material 2 [file 420_2024_2083_MOESM2_ESM.png]

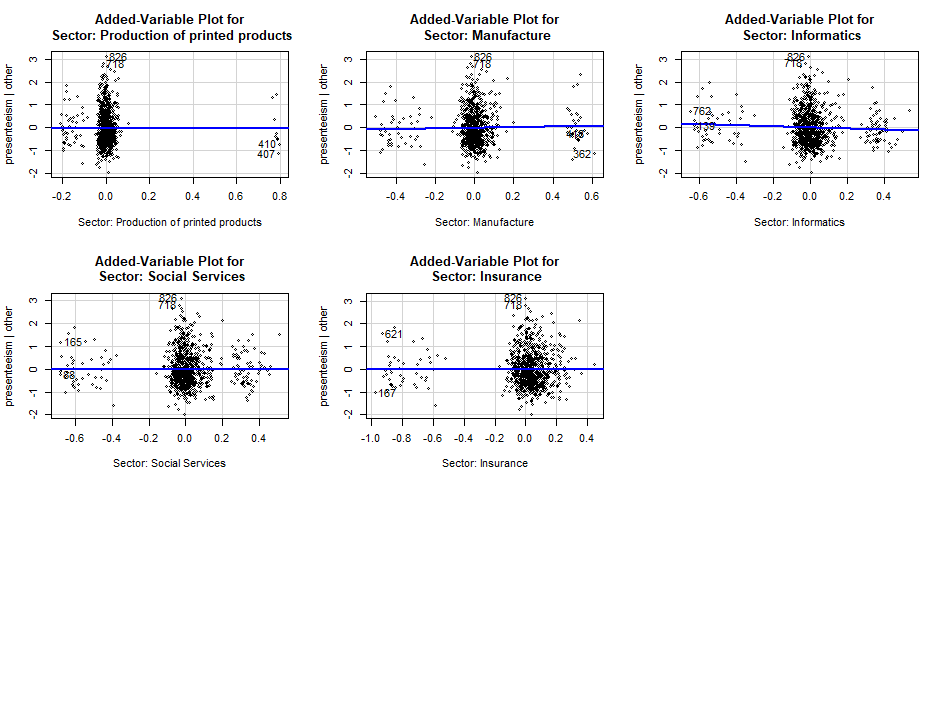

Supplement: Supplementary file 3 — Supplementary Material 3 [file 420_2024_2083_MOESM3_ESM.png]
